# Beyond protein synthesis: non-translational functions of threonyl-tRNA synthetases

**DOI:** 10.1042/BST20230506

**Published:** 2024-03-13

**Authors:** Pallob Barai, Jie Chen

**Affiliations:** Department of Cell and Developmental Biology, University of Illinois at Urbana-Champaign, Champaign, IL, USA

**Keywords:** aminoacyl-trna synthetase, non-canonical, non-translational, threonyl-tRNA synthetase

## Abstract

Aminoacyl-tRNA synthetases (AARSs) play an indispensable role in the translation of mRNAs into proteins. It has become amply clear that AARSs also have non-canonical or non-translational, yet essential, functions in a myriad of cellular and developmental processes. In this mini-review we discuss the current understanding of the roles of threonyl-tRNA synthetase (TARS) beyond protein synthesis and the underlying mechanisms. The two proteins in eukaryotes — cytoplasmic TARS1 and mitochondrial TARS2 — exert their non-canonical functions in the regulation of gene expression, cell signaling, angiogenesis, inflammatory responses, and tumorigenesis. The TARS proteins utilize a range of biochemical mechanisms, including assembly of a translation initiation complex, unexpected protein–protein interactions that lead to activation or inhibition of intracellular signaling pathways, and cytokine-like signaling through cell surface receptors in inflammation and angiogenesis. It is likely that new functions and novel mechanisms will continue to emerge for these multi-talented proteins.

## Aminoacyl-tRNA synthetases

Aminoacyl-tRNA synthetases (AARSs) are a family of essential enzymes that catalyze aminoacylation — ligation of amino acids to their cognate tRNAs, thus creating substrates for protein synthesis [1,2]. There are two sets of these enzymes in eukaryotes — cytoplasmic and mitochondrial, encoded by distinct genes except for two of them (see below). There are various nomenclatures for this family of proteins. In this review we will follow the Human Genome Organisation gene nomenclature and use single-letter amino acid codes for the gene and protein names in higher organisms (e.g. TARS1 for cytoplasmic threonyl-tRNA synthetase and TARS2 for the mitochondrial counterpart). However, for the bacterial and yeast proteins we will follow the convention in the field and use three-letter amino acid codes (e.g. ThrRS for bacterial threonyl tRNA-synthetase). The human genome contains 37 genes encoding AARS proteins, with 18 in the cytoplasm, 17 in the mitochondria, and 2 in both compartments (KARS and GARS).

AARSs attach each amino acid to the 3′ end of its cognate tRNA, forming an ester linkage with the terminal adenosine [1,2]. In addition to accurate recognition of the tRNA, precise non-covalent binding of the cognate amino acid by the AARS is essential to prevent errors in protein synthesis [2]. However, AARSs can undergo misacylation under certain stress conditions like oxidative stress [3]. To circumvent the effects of misacylation, most AARSs possess proofreading and editing capabilities, which can occur either before the amino acid transfer step, involving hydrolysis of aminoacyl adenylate, or post-transfer by deacylation of mischarged aa-tRNA [4–6].

Despite the limited primary sequence similarities within the family, AARSs can be categorized into Class I and Class II based on distinct sequence motifs at the active sites [7]. Class I AARSs are primarily monomeric proteins with the conserved sequence motifs ‘HIGH’ and ‘KMSKS’, and a Rossmann dinucleotide binding domain. Class II AARSs are functional dimers or tetramers with the conserved Motifs 1, 2, 3 and an antiparallel β-fold in the active site. Class I enzymes transfer amino acids onto the 2′-OH group of the terminal adenosine of tRNA, whereas Class II enzymes mostly transfer amino acids to the 3′-OH.

## AARSs have non-translational functions

While decades of studies have led to deep understanding of the house-keeping function and mechanisms of AARSs, more recent years have seen a surge in discoveries of non-canonical or non-translational functions for many cytoplasmic (as well as mitochondrial) AARSs in a broad range of cellular regulations [8–11]. For instance, AARSs are found to regulate gene expression at all levels, including transcription, splicing, and translation (non-canonically), via diverse mechanisms. Through dysregulation in their translational and non-translational functions, AARSs are known to be associated with many human diseases including but not limited to neuropathies, chronic inflammatory diseases, and cancer [10,12–16].

Almost all the eukaryotic AARSs contain ‘new domains’ (distinct for each AARS) appended to the catalytic core, the acquisition of which coincided with the emergence of increasing complexity of eukaryotic organisms [9]. Some of these newly appended domains are found in multiple AARSs, including leucine zipper (LZ), glutathione *S*-transferase, WHEP (named after its presence in WARS1, HARS1, and EPRS1), whereas other domains are unique to individual AARSs (e.g. UNE-L for LARS1, UNE-S for SARS1, etc.). Once acquired, the new domains became conserved in higher organisms, suggesting that AARSs may perform essential, non-translational functions through these domains. Indeed, there are numerous cases of non-translational functions involving the new domains of AARSs [8,9]. For example, the WHEP domain of WARS1 is involved in the activation of p53 in the nucleus, whereas the multiple WHEP domains in EPRS1 play a critical role in γ­interferon activated inhibition of translation (reviewed in [10,17]). Along with the emergence of the new domains in AARSs, higher eukaryotes evolved to have a multi-synthetase complex (MSC). The mammalian MSC contains eight cytoplasmic synthetase proteins — DARS1, EPRS1 (glutamyl- and prolyl-tRNA synthetase), KARS1, IARS1, LARS1, MARS1, QARS1, and RARS1, and three scaffold proteins — AARS-interacting multifunctional protein 1, 2 and 3 (AIMP1, AIMP2, and AIMP3). The MSC is believed to facilitate the translational function of the AARSs, as well as serving as a depot for AARSs with non-canonical functions [18–22].

In many cases the catalytic core of AARSs can also confer non-catalytic functions. For instance, the amino acid binding sites of several AARSs are utilized for non-translational functions, such as leucine-sensing by LARS1 in the activation of mammalian target of rapamycin complex 1 (mTORC1) signaling, glutamine regulation of apoptosis signal-regulating kinase 1 signaling through QARS1, and binding to VE-cadherin by an extracellular N-terminal fragment of WARS1 to exert anti-angiogenesis effects (reviewed in [23]). The readers are referred to excellent reviews on non-translational functions of AARSs cited above [8–11]. In this mini-review we focus on TARS. Recent developments and future prospects in uncovering the non-translational functions of TARS are discussed.

## TARS

**Figure 1.**
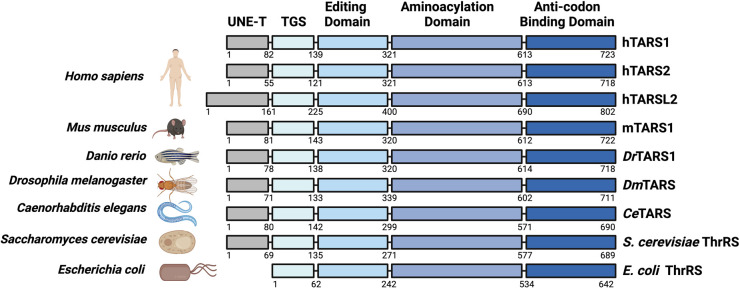
Schematic representation of the domain structures of TARS across species. Amino acid residue numbers defining the domains are obtained from Uniprot (https://www.uniprot.org/) and/or determined by sequence alignment in Clustal Omega (https://www.ebi.ac.uk/jdispatcher/msa/clustalo). For ease of alignment, not all domains are drawn to scale. Figure created with BioRender.com.

TARS is a class II dimeric enzyme, further categorized as a subclass IIa protein [7,24]. The first structural analyses of TARS were conducted on *Escherichia coli* ThrRS. The dimeric core of *E. coli* ThrRS is formed by the catalytic and C-terminal anticodon binding domains, while the N-terminal editing domain is situated on the opposite side of the core [25,26]. One copy of tRNA interacts with both monomers, engaging the catalytic domain, C-terminus, and editing domain. The very N-terminus of *E. coli* ThrRS is the TGS domain (named after its presence in TARS, GTPase, and SpoT) [27], which is conserved among prokaryotes and eukaryotes. In the course of evolution eukaryotic TARS gained an N-terminal extension, the UNE-T domain. Despite bacterial origin of the mitochondrion, the mammalian mitochondrial TARS2 exhibits greater sequence similarities to cytoplasmic TARS1 than to bacterial ThrRS [28,29]. Like its cytoplasmic counterpart, the mammalian mitochondrial TARS2 has an editing activity that is critical for translation fidelity and important for mitochondrial function and cell proliferation [30]. Vertebrates express an additional TARS-like protein named TARSL2, which exhibits marked sequence similarities to TARS1 except for a larger N-terminal domain [31] (Figure 1).

Like other AARSs, TARS can perform various non-canonical or non-translational functions involved in gene expression, cell signaling, angiogenesis, inflammation, and tumorigenesis as discussed below. The functional diversification of TARS is evident across both higher- and lower-order organisms.

## TARS in non-canonical regulation of translation

As one of the earliest non-canonical functions identified, negative regulation of the translation of its own mRNA by *E. coli* ThrRS exemplifies how an AARS can achieve autoregulation [32–36]. ThrRS binds to regions upstream of translational start site of its mRNA and subsequently inhibits translation by preventing binding of the 30S ribosome. The control element in the ThrRS leader mRNA, known as operator, consists of four structural domains: the ribosome binds domain I and 3′ part of the linker domain III; domains II and IV create two hairpins, recognized by ThrRS due to their structural similarity to the anticodon loop of tRNA^Thr^. Replacing the anticodon-like sequence in the ThrRS mRNA with that of tRNA^Met^ in the operator switches the regulation of translation from ThrRS to MetRS [37,38], suggesting that this autoregulatory mechanism may apply to other AARSs.

**Figure 2.**
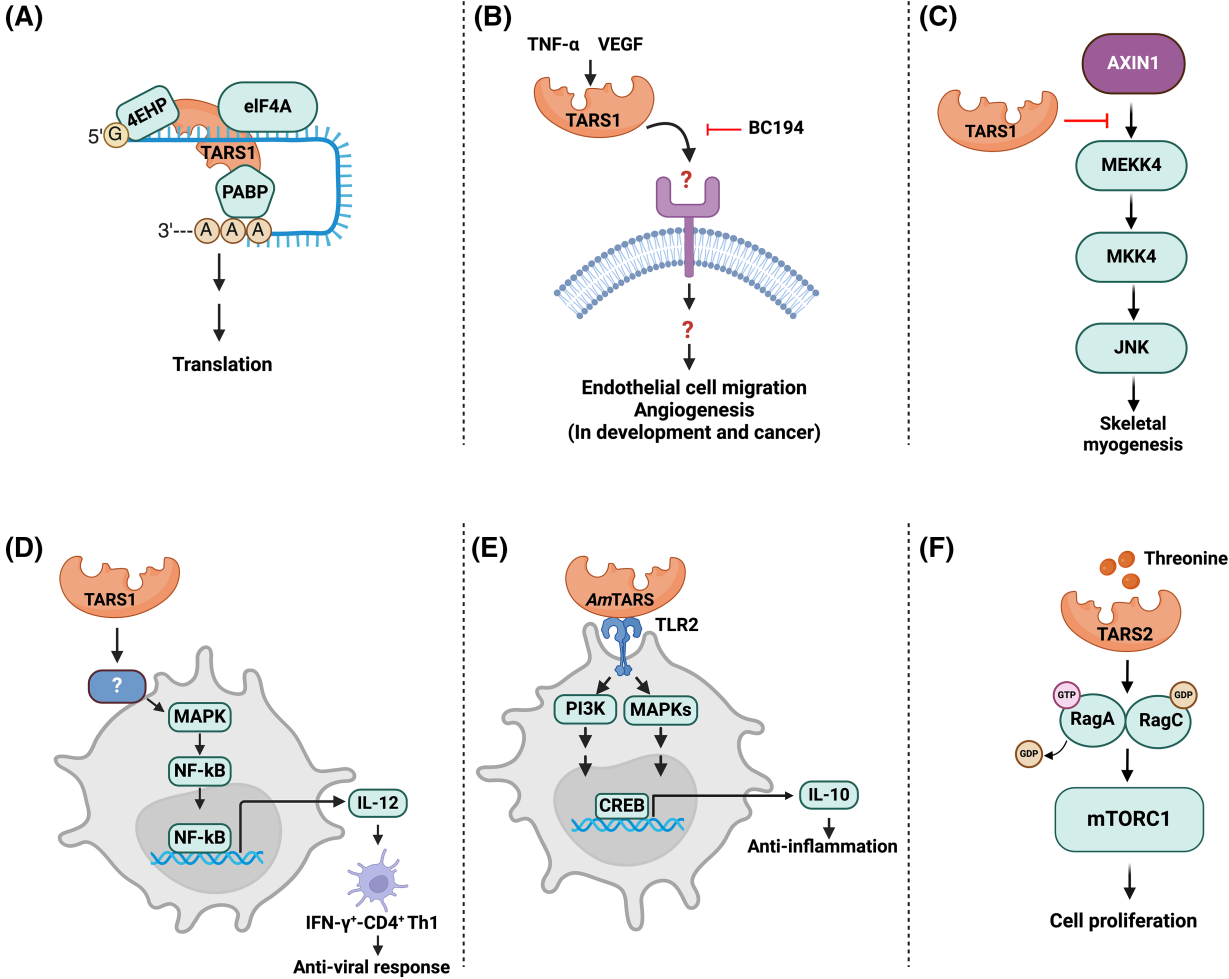
Non-canonical functions of TARS. (**A**) TARS1 assembles a translation initiation complex involving 4EHP, eIF4A and PABP. (**B**) TNFα or VEGF induces secretion of TARS1, which through an unknown receptor regulates endothelial cell migration and angiogenesis (potentially relevant in both vascular development and cancer). (**C**) TARS1 negatively regulates skeletal myogenesis by inhibiting JNK signaling through interaction with Axin1. (**D**) Extracellular TARS1 induces dendritic cell maturation and activation through the activation of MAP kinases ERK and JNK followed by NF-κB activation. Activated dendritic cells produce IL-12, which promotes Th1 anti-viral response. (**E**) *Am*TARS binds TLR2 on macrophages and activates MAPK (ERK and p38) and PI3K signaling, resulting in activation of CREB and subsequent production of the anti-inflammatory IL-10. (**F**) TARS2 binds and activates the RagA/C dimer in response to threonine, and subsequently activates mTORC1 in the regulation of cell proliferation. Figure created with BioRender.com.

More recently, Jeong et al. [39] uncovered an unexpected function of human TARS1 in assembling a translation initiation machinery. Through affinity purification and mass spectrometry analysis, TARS1 was found to interact with 4EHP (eIF4E2), a homolog of the 5′cap-binding protein eIF4E. The UNE-T domain of TARS1 directly interacts with cap-bound 4EHP and, furthermore, TARS1 also interacts with eIF4A and the polyA-binding protein PABP, but not eIF4G [39]. Hence, TARS1 mimics eIF4G as a scaffold to assemble a cap-dependent translation initiation complex containing 4EHP, eIF4A, and PABP (Figure 2A). This TARS1-mediated translation initiation machinery most likely regulates a subset of mRNAs specifically recognized by 4EHP, and many of those mRNAs encode proteins that control biological processes specific for vertebrates, such as neuronal, skeletal, and vascular development. Vascular endothelial growth factor (VEGF) expression at the translational level was shown to be dependent on TARS1–4EHP interaction, and so was angiogenesis in human cells and zebrafish [39]. Indeed, the TARS1–4EHP interaction is only observed in vertebrates, consistent with sequence divergence found in the UNE-T domain as well as in 4EHP's TARS1-binding site in lower organisms [39].

## Extracellular TARS1 and angiogenesis

The first clue of TARS involvement in angiogenesis came from the strong anti-angiogenic effect of borrelidin [40], a potent non-competitive TARS inhibitor isolated from *Streptomyces rochei* [41,42]. However, borrelidin interacts with the threonine-binding pocket in the TARS1 active site, and consequently elicits amino acid starvation response and apoptosis in endothelial cells [43]. It is therefore not possible to uncouple the anti-angiogenic effect of this inhibitor from its inhibition of TARS catalytic activity or its general toxicity. More recent studies using the borrelidin analog BC194, which has a drastically weakened affinity for the threonine binding site while still exhibiting a full anti-angiogenic activity in human cells and zebrafish [44,45], have provided more definitive evidence for a non-canonical function of TARS1 in angiogenesis regulation.

Several mammalian AARSs are secreted either as intact proteins, fragments, or splice variants, and they can act as extracellular signaling proteins [46]. Williams et al. found TARS1 to be secreted by human umbilical vein endothelial cells in response to stimulation by tumor necrosis factor-α (TNF-α) or VEGF [47]. Extracellular TARS1 is shown to stimulate angiogenesis both *in vitro* and *in vivo*, and TARS1 likely exerts this angiogenic effect by promoting endothelial cell migration (Figure 2B). A mutant of TARS1 (R442A) that no longer has any aminoacylation activity is fully effective in stimulating angiogenesis, further validating the non-translational nature of this function [47]. It remains to be determined in what form TARS1 is secreted and which receptor mediates the signaling by extracellular TARS1 in the regulation of angiogenesis.

Another connection between TARS1 and angiogenesis came from the characterization of a zebrafish mutant line (cq16) that displayed abnormal branching and patterning of brain vascular network during embryonic development [48]. VEGF-A expression was increased in the mutant embryo, and pharmacological inhibition of VEGF receptor signaling suppressed the vascular phenotype. Interestingly, the cq16 gene was found to encode TARS1 with a missense mutation in the catalytic domain. However, since the biochemical effect of this mutation has not been characterized, it is not possible to speculate whether the wild-type TARS1 stimulates or inhibits angiogenesis, or whether TARS1 exerts its function canonically or non-canonically.

## TARS1 inhibition of JNK signaling in myogenic differentiation

A few years ago, our group reported a non-translational role of LARS1 in regulating skeletal muscle differentiation and regeneration through the mTORC1 pathway [49]. Those findings prompted us to ask whether any other AARSs may also regulate skeletal myogenesis. An RNAi screen of cytoplasmic AARSs in the mouse myoblast C2C12 cells led to the identification of TARS1 as a potential regulator of myogenic differentiation. Through knockdown and overexpression experiments TARS1 is found to negatively control myoblast differentiation in culture and injury-induced muscle regeneration in mice [50]. This regulation by TARS1 is independent of global protein synthesis, and the catalytic activity of TARS1 is dispensable, indicating a non-translational function. To dissect the mechanism of this novel function, we surveyed major signaling pathways known to regulate myogenesis, and JNK signaling emerged to be specifically down-regulated by TARS1 in myoblasts. We further discovered that TARS1 physically interacted with Axin1, which disrupted Axin1 interaction with MEKK4, resulting in the inhibition of the Axin1–MEKK4–MKK4 pathway upstream of JNK [50] (Figure 2C). A positive role of JNK in regulating myogenic differentiation has been previously established [51,52].

Strikingly, the N-terminal UNE-T and TGS domains of TARS1 together are both necessary and sufficient for the inhibition of myogenic differentiation [50]. It is interesting to note that a naturally occurring splice variant of human TARS1 encodes an N-terminal fragment of the protein that encompasses UNE-T and TGS domains [53]. It is not known whether this splice variant is expressed in mouse myoblasts and, if so, whether its protein product is involved in myogenic regulation. Meanwhile, a potential link between this N-terminal protein product and JNK signaling in other cell types known to express the splice variant [53] will also be worthy of attention in future investigations.

## TARS in immunity and inflammation

While pathogen AARSs often elicit immune responses and can serve as anti-infective targets, mammalian AARSs are involved in immune cell development as well as signaling in immune responses [54,55]. TARS1 is one of several AARSs found to be up-regulated in expression in response to viral infection [54]. In a recent report, Jung et al. [56] showed that extracellular TARS1 induced the maturation and activation of dendritic cells (DCs), likely through the activation of the MAP kinases ERK and JNK, and ultimately NF-κB activation. Treatment with extracellular TARS1 led to enhanced DC secretion of the cytokine IL-12, which in turn induced the polarization of CD4^+^ T cells into Th1 cells (IFNγ^+^CD4^+^) [56] (Figure 2D). Notably, *in vivo* relevance of these findings was confirmed by the observation that infusion of TARS1-treated DCs induced a Th1 response and enhanced the antiviral effect of the DCs [56]. TARS1 presumably interacts with a yet-to-be-identified cell surface receptor to perform the function described here. These findings may have important implications in the association of TARS1 with autoimmune diseases such as polymyositis and dermatomyositis, in which TARS1 is the target of the autoantibody PL-7 [57].

Most recently, Kim et al. [58] have reported that the gut-associated bacteria *Akkermansia muciniphila* secrete TARS (*Am*TARS) to modulate immune homeostasis. They show that extracellular *Am*TARS induces the polarization of M2 macrophages and subsequent production of the anti-inflammatory cytokine IL-10, which ameliorates the inflammatory effects of colitis in mice [58]. The authors further identify amino acid regions unique to *Am*TARS that are responsible for interacting with the toll-like receptor TLR2. By engaging TLR2, *Am*TARS acts as a ligand for the receptor to activate intracellular signaling pathways including the MAP kinases ERK and p38, as well as PI3K–AKT, which converge on CREB activation in the nucleus [58] (Figure 2E). TLR2 represents the first cell surface receptor to be identified to receive signal from an extracellular TARS. Although mammalian TARS1 is unlikely to utilize the same receptor due to its lack of sequences homologous to those in *Am*TARS mediating TLR2 interaction, the work by Kim et al. should inspire others to pursue the elusive receptor for mammalian TARS1 in the regulation of angiogenesis or immune cell development.

## TARS2 regulation of mTORC1 signaling

mTORC1 is a master regulator of cell growth and proliferation by mediating and integrating environmental cues, including nutrient availability, growth factor signals, energy levels, and various types of stress [59–61]. mTORC1 has been known to transduce the signals of cellular amino acid sufficiency through a multitude of proteins as sensors specifically for leucine, glutamine, arginine, and methionine [62–69]. These amino acid sensors regulate the Rag small G proteins (RagA/B/C/D), which functions as a heterodimer to activate mTORC1 on the surface of the lysosome [70]. More recently, Kim et al. [71] discovered that the mitochondrial TARS2 physically associates with the protein complex responsible for amino acid-dependent mTORC1 activation at the lysosome. The authors went on to show that TARS2 is necessary for mTORC1 translocation to lysosomes and activation in response to threonine stimulation, and that TARS2 performs this role by binding to inactive RagC and promoting RagA GTP-loading (Figure 2F) [71]. Exactly how TARS2 modulates guanine nucleotide exchange of RagA is not known, but the collective evidence presented by Kim et al. supports a role of TARS2 in mediating the sensing of cellular threonine by mTORC1 in the regulation of cell proliferation. TARS2 represents another example of AARSs utilizing their specific amino acid binding to confer non-translational functions.

## TARS in cancer

Genomic and transcriptomic analyses of cancer databases have revealed a strong cancer-associated profile for human AARSs as a family [72]. Individual AARSs have distinct profiles, some resembling tumor suppressors and others oncogenes [73]. Although the elevated expression levels of several AARSs in cancer could reflect a heightened demand for protein synthesis in cancer cells, non-translational functions of AARSs are implicated by the diverse genomic and transcriptomic profiles of AARSs in cancer. Indeed, several AARSs have been demonstrated to have direct or indirect roles in tumorigenesis independent of protein synthesis [72,74].

A potential link of TARS1 to cancer was speculated based on the epidemiological connection between myositis and cancer [75]. TARS1 is one of the most highly expressed AARSs in cancer when all cancer types are taken into consideration (our unpublished observation and [73]). More importantly, high expression levels of TARS1 significantly correlate with poor patient survival in several types of cancer including breast, lung, ovarian, liver, and pancreatic cancer (https://kmplot.com [76]). A canonical function of TARS1 has been suggested in pancreatic cancer, where the overexpression of Mucin1 (MUC1) and cell migration are dependent on high levels of TARS1 due to an unusually high number of threonine residues in the amino acid sequence of MUC1 [77]. It is not clear whether this mechanism occurs in other types of cancer.

Following the discovery of an angiogenic function of extracellular TARS1 [47], Wellman et al. [78] examined a potential role of TARS1 in the highly angiogenic ovarian cancer. Indeed, they have found that ovarian cancer cells secrete TARS1, and patient serum TARS1 levels correlate with TARS1 expression in tumor samples. Furthermore, overexpression of TARS1 strongly correlates with advanced stages of the disease as well as the angiogenic marker VEGF. However, paradoxically, in late-stage disease an inverse correlation is found between TARS1 expression and patient mortality [78]. Wellman et al. propose a complex role for TARS1 in ovarian cancer that involves the tumor microenvironment, angiogenesis, and immune cell response.

Elevated expression of mitochondrial TARS2 is also found to correlate with patient mortality in non-small cell lung adenocarcinoma (LUAD). Tian et al. recently showed that knockdown of TARS2 in LUAD cells inhibited proliferation and increased mitochondrial reactive oxygen species-induced apoptosis, and that TARS2 knockdown also suppressed xenograft tumor growth *in vivo* [79]. However, mTORC1 signaling, a master regulator of cell growth/proliferation and reported to be activated by TARS2 [71], was not examined in the study. Future investigation will be necessary to probe the mechanism of TARS2 action in LUAD.

## Function of TARSL2

A unique aspect of TARS is the duplication of the TARS1 gene in higher eukaryotes, resulting in TARSL2 [31]. Characterization of TARSL2 by En-Duo Wang and colleagues in recent years has shed significant light on this curious homolog. As expected from its high sequence similarity to TARS1, TARSL2 has aminoacylation and editing activities [80]. However, TARSL2 appears to be dispensable for protein synthesis, as deletion of the *Tarsl2* gene does not affect the aminoacylation of tRNA^Thr^ [81]. While knockout of *Tars1* results in embryonic lethality in mice, *Tarsl2* knockout mice appear normal at birth [81]. Nevertheless, growth retardation becomes evident after 3 weeks of age in the *Tarsl2* knockout mice, with defects observed in bone development and skeletal muscle formation. Remarkably, the *Tarsl2* knockout mice are leaner than WT, with enhanced glucose and lipid metabolism [81]. Future investigation will be necessary to characterize the mechanism of TARSL2 action that underlies those striking phenotypes.

Interestingly, biochemical evidence suggests that TARSL2 may be a component of the MSC [82–84]. The extended N-terminus, which contains two LZs, can interact with other components of the MSC to facilitate the incorporation of TARSL2 in the complex [83]. However, the functional relevance of TARSL2's presence in the MSC is not clear. Knockout of *Tarsl2* does not affect the integrity of MSC, ruling out a role of scaffolding for TARSL2 in the MSC [81]. Of note, TARS1 and TARSL2 can form a heterodimer when overexpressed in cells [83]. It is also noteworthy that the catalytic activity of TARSL2 has been maintained throughout evolution. All these biochemical features of TARSL2 should be kept in mind during future investigation of this fascinating protein.

## Concluding remarks and future prospects

Many AARSs continue to surprise us with new non-canonical functions, and TARS is a striking example. The range of novel functions assigned to TARS1 and TARS2 discussed above may represent only a fraction of what these house-keeping proteins can do. While the regulation of some processes is unique to TARS (e.g. TARS2 assembly of the translational initiation machinery; threonine-dependent mTORC1 activation by TARS1), other aspects of biology can involve multiple AARSs although each with a distinct mechanism (e.g. TARS1 and LARS1 in skeletal myogenesis). It is remarkable that a family of proteins with well-conserved functional domains can device such wide-raging biochemical mechanisms to exert non-canonical regulation. The ‘new domains’ unique to each AARS certainly contribute to the diversity, but even the conserved catalytic domains can have specific non-canonical functions in individual AARSs. Cellular contexts, such as the presence of cofactors and/or distinct subcellular localization, can confer cell type-specific functions for TARS, as is likely the case for TARS1 regulation of JNK signaling exclusively in muscle cells. These context-specific regulatory mechanisms, as well as yet-to-be-discovered new functions, warrant significant future research efforts. The involvement of TARS in muscle regeneration, angiogenesis, immunity, and cancer promises tremendous potential in future therapeutic development.

## Perspectives

- Like many other AARSs, TARS proteins play essential roles in a wide range of cellular and developmental processes, which are independent of their canonical function in protein synthesis.
- The two eukaryotic proteins, cytoplasmic TARS1 and mitochondrial TARS2, are found to non-canonically or non-translationally regulate gene expression, cell signaling, angiogenesis, inflammatory responses, and tumorigenesis, through diverse biochemical mechanisms.
- Dissecting mechanisms underlying the currently known TARS functions and identifying additional biological processes involving TARS will deepen our fundamental understanding of biological regulation and facilitate future therapeutic exploration against several human diseases including cancer.

## Abbreviations

AARS: aminoacyl-tRNA synthetase
*Am*TARS: *Akkermansia muciniphila* secrete TARS
DC: dendritic cell
LUAD: lung adenocarcinoma
LZ: leucine zipper
MSC: multi-synthetase complex
mTORC1: mammalian target of rapamycin complex 1
MUC1: Mucin1
PABP: polyA-binding protein
TNF-α: tumor necrosis factor-α
VEGF: vascular endothelial growth factor

## References

[1] Schimmel, P.R. and Söll, D. (1979) Aminoacyl-tRNA synthetases: general features and recognition of transfer RNAs. Annu. Rev. Biochem. 48, 601–648 10.1146/annurev.bi.48.070179.003125 382994

[2] Ibba, M. and Soll, D. (2000) Aminoacyl-tRNA synthesis. Annu. Rev. Biochem. 69, 617–650 10.1146/annurev.biochem.69.1.617 10966471

[3] Ribas de Pouplana, L., Santos, M.A.S., Zhu, J.-H., Farabaugh, P.J. and Javid, B. (2014) Protein mistranslation: friend or foe? Trends Biochem. Sci. 39, 355–362 10.1016/j.tibs.2014.06.002 25023410

[4] Perona, J.J. and Gruic-Sovulj, I. (2014) Synthetic and editing mechanisms of aminoacyl-tRNA synthetases. Top. Curr. Chem. 344, 1–41 10.1007/128_2013_456 23852030

[5] Advani, V.M. and Ivanov, P. (2019) Translational control under stress: reshaping the translatome. BioEssays 41, e1900009 10.1002/bies.201900009 31026340 PMC6541386

[6] Steiner, R.E. and Ibba, M. (2019) Regulation of tRNA-dependent translational quality control. IUBMB Life 71, 1150–1157 10.1002/iub.2080 31135095

[7] Eriani, G., Delarue, M., Poch, O., Gangloff, J. and Moras, D. (1990) Partition of tRNA synthetases into two classes based on mutually exclusive sets of sequence motifs. Nature 347, 203–206 10.1038/347203a0 2203971

[8] Guo, M. and Schimmel, P. (2013) Essential nontranslational functions of tRNA synthetases. Nat. Chem. Biol. 9, 145–153 10.1038/nchembio.1158 23416400 PMC3773598

[9] Guo, M., Yang, X.L. and Schimmel, P. (2010) New functions of aminoacyl-tRNA synthetases beyond translation. Nat. Rev. Mol. Cell Biol. 11, 668–674 10.1038/nrm2956 20700144 PMC3042954

[10] Kwon, N.H., Fox, P.L. and Kim, S. (2019) Aminoacyl-tRNA synthetases as therapeutic targets. Nat. Rev. Drug Discov. 18, 629–650 10.1038/s41573-019-0026-3 31073243

[11] Yao, P., Poruri, K., Martinis, S.A. and Fox, P.L. (2014) Non-catalytic regulation of gene expression by aminoacyl-tRNA synthetases. In Aminoacyl-tRNA Synthetases in Biology and Medicine (Kim, S., ed.), pp. 167–187, Springer, Dordrecht, Netherlands 10.1007/128_2013_422 23536244

[12] Park, S.G., Schimmel, P. and Kim, S. (2008) Aminoacyl tRNA synthetases and their connections to disease. Proc. Natl Acad. Sci. U.S.A. 105, 11043–11049 10.1073/pnas.0802862105 18682559 PMC2516211

[13] Kim, D., Kwon, N.H. and Kim, S. (2014) Association of aminoacyl-tRNA synthetases with cancer. Top. Curr. Chem. 344, 207–245 10.1007/128_2013_455 23818134

[14] Yao, P. and Fox, P.L. (2013) Aminoacyl-tRNA synthetases in medicine and disease. EMBO Mol. Med. 5, 332–343 10.1002/emmm.201100626 23427196 PMC3598075

[15] Wei, N., Zhang, Q. and Yang, X.L. (2019) Neurodegenerative Charcot-Marie-Tooth disease as a case study to decipher novel functions of aminoacyl-tRNA synthetases. J. Biol. Chem. 294, 5321–5339 10.1074/jbc.REV118.002955 30643024 PMC6462521

[16] Turvey, A.K., Horvath, G.A. and Cavalcanti, A.R.O. (2022) Aminoacyl-tRNA synthetases in human health and disease. Front. Physiol. 13, 1029218 10.3389/fphys.2022.1029218 36330207 PMC9623071

[17] Lee, E.Y., Hwang, J. and Kim, M.H. (2023) Phosphocode-dependent glutamyl-prolyl-tRNA synthetase 1 signaling in immunity, metabolism, and disease. Exp. Mol. Med. 55, 2116–2126 10.1038/s12276-023-01094-x 37779151 PMC10618286

[18] Ray, P.S., Arif, A. and Fox, P.L. (2007) Macromolecular complexes as depots for releasable regulatory proteins. Trends Biochem. Sci. 32, 158–164 10.1016/j.tibs.2007.02.003 17321138

[19] Guo, M. and Yang, X.L. (2014) Architecture and metamorphosis. Top. Curr. Chem. 344, 89–118 10.1007/128_2013_424 23536245 PMC3864538

[20] Hyeon, D.Y., Kim, J.H., Ahn, T.J., Cho, Y., Hwang, D. and Kim, S. (2019) Evolution of the multi-tRNA synthetase complex and its role in cancer. J. Biol. Chem. 294, 5340–5351 10.1074/jbc.REV118.002958 30782841 PMC6462501

[21] Khan, K., Gogonea, V. and Fox, P.L. (2022) Aminoacyl-tRNA synthetases of the multi-tRNA synthetase complex and their role in tumorigenesis. Transl. Oncol. 19, 101392 10.1016/j.tranon.2022.101392 35278792 PMC8914993

[22] Kim, M.H. and Kim, S. (2020) Structures and functions of multi-tRNA synthetase complexes. Enzymes 48, 149–173 10.1016/bs.enz.2020.06.008 33837703

[23] Yu, Y.C., Han, J.M. and Kim, S. (2021) Aminoacyl-tRNA synthetases and amino acid signaling. Biochim. Biophys. Acta Mol. Cell Res. 1868, 118889 10.1016/j.bbamcr.2020.118889 33091505

[24] Cusack, S. (1995) Eleven down and nine to go. Nat. Struct. Biol. 2, 824–831 10.1038/nsb1095-824 7552701

[25] Sankaranarayanan, R., Dock-Bregeon, A.-C., Romby, P., Caillet, J., Springer, M., Rees, B. et al. (1999) The structure of threonyl-tRNA synthetase-tRNAThr complex enlightens its repressor activity and reveals an essential zinc ion in the active site. Cell 97, 371–381 10.1016/s0092-8674(00)80746-1 10319817

[26] Dock-Bregeon, A.-C., Sankaranarayanan, R., Romby, P., Caillet, J., Springer, M., Rees, B. et al. (2000) Transfer RNA–mediated editing in threonyl-tRNA synthetase. Cell 103, 877–884 10.1016/s0092-8674(00)00191-4 11136973

[27] Wolf, Y.I., Aravind, L., Grishin, N.V. and Koonin, E.V. (1999) Evolution of aminoacyl-tRNA synthetases–analysis of unique domain architectures and phylogenetic trees reveals a complex history of horizontal gene transfer events. Genome Res. 9, 689–710 10.1101/gr.9.8.689 10447505

[28] Wang, Y., Zhou, X.-L., Ruan, Z.-R., Liu, R.-J., Eriani, G. and Wang, E.-D. (2016) A human disease-causing point mutation in mitochondrial threonyl-tRNA synthetase induces both structural and functional defects. J. Biol. Chem. 291, 6507–6520 10.1074/jbc.M115.700849 26811336 PMC4813579

[29] Peng, G.X., Mao, X.L., Cao, Y., Yao, S.Y., Li, Q.R., Chen, X. et al. (2022) RNA granule-clustered mitochondrial aminoacyl-tRNA synthetases form multiple complexes with the potential to fine-tune tRNA aminoacylation. Nucleic Acids Res. 50, 12951–12968 10.1093/nar/gkac1141 36503967 PMC9825176

[30] Zheng, W.Q., Zhang, J.H., Li, Z.H., Liu, X., Zhang, Y., Huang, S. et al. (2023) Mammalian mitochondrial translation infidelity leads to oxidative stress-induced cell cycle arrest and cardiomyopathy. Proc. Natl Acad. Sci. U.S.A. 120, e2309714120 10.1073/pnas.2309714120 37669377 PMC10500172

[31] Zhou, X.L., Ruan, Z.R., Huang, Q., Tan, M. and Wang, E.D. (2013) Translational fidelity maintenance preventing Ser mis-incorporation at Thr codon in protein from eukaryote. Nucleic Acids Res. 41, 302–314 10.1093/nar/gks982 23093606 PMC3592468

[32] Springer, M., Graffe, M., Dondon, J. and Grunberg-Manago, M. (1989) tRNA-like structures and gene regulation at the translational level: a case of molecular mimicry in *Escherichia coli*. EMBO J. 8, 2417–2424 10.1002/j.1460-2075.1989.tb08372.x 2676521 PMC401186

[33] Butler, J.S., Springer, M., Dondon, J. and Grunberg-Manago, M. (1986) Posttranscriptional autoregulation of *Escherichia coli* threonyl tRNA synthetase expression in vivo. J. Bacteriol. 165, 198–203 10.1128/jb.165.1.198-203.1986 3510186 PMC214389

[34] Moine, H., Ehresmann, B., Romby, P., Ebel, J.P., Grunberg-Manago, M., Springer, M. et al. (1990) The translational regulation of threonyl-tRNA synthetase. Functional relationship between the enzyme, the cognate tRNA and the ribosome. Biochim. Biophys. Acta 1050, 343–350 10.1016/0167-4781(90)90192-5 2207165

[35] Torres-Larios, A., Dock-Bregeon, A.C., Romby, P., Rees, B., Sankaranarayanan, R., Caillet, J. et al. (2002) Structural basis of translational control by *Escherichia coli* threonyl tRNA synthetase. Nat. Struct. Biol. 9, 343–347 10.1038/nsb789 11953757

[36] Moine, H., Romby, P., Springer, M., Grunberg-Manago, M., Ebel, J.P., Ehresmann, B. et al. (1990) *Escherichia coli* threonyl-tRNA synthetase and tRNA(Thr) modulate the binding of the ribosome to the translational initiation site of the thrS mRNA. J. Mol. Biol. 216, 299–310 10.1016/s0022-2836(05)80321-3 2254931

[37] Graffe, M., Dondon, J., Caillet, J., Romby, P., Ehresmann, C., Ehresmann, B. et al. (1992) The specificity of translational control switched with transfer RNA identity rules. Science 255, 994–996 10.1126/science.1372129 1372129

[38] Romby, P., Brunel, C., Caillet, J., Springer, M., Grunberg-Manago, M., Westhof, E. et al. (1992) Molecular mimicry in translational control of *E. coli* threonyl-tRNA synthetase gene. Competitive inhibition in tRNA aminoacylation and operator-repressor recognition switch using tRNA identity rules. Nucleic Acids Res. 20, 5633–5640 10.1093/nar/20.21.5633 1280807 PMC334396

[39] Jeong, S.J., Park, S., Nguyen, L.T., Hwang, J., Lee, E.Y., Giong, H.K. et al. (2019) A threonyl-tRNA synthetase-mediated translation initiation machinery. Nat. Commun. 10, 1357 10.1038/s41467-019-09086-0 30902983 PMC6430810

[40] Wakabayashi, T., Kageyama, R., Naruse, N., Tsukahara, N., Funahashi, Y., Kitoh, K. et al. (1997) Borrelidin is an angiogenesis inhibitor; disruption of angiogenic capillary vessels in a rat aorta matrix culture model. J. Antibiot. 50, 671–676 10.7164/antibiotics.50.671 9315080

[41] Berger, J., Jampolsky, L.M. and Goldberg, M.W. (1949) Borrelidin, a new antibiotic with antiborrelia activity and penicillin enhancement properties. Arch. Biochem. 22, 476–478 18134558

[42] Anderton, K. and Rickards, R.W. (1965) Some structural features of borrelidin, an anti-viral antibiotic. Nature 206, 269 10.1038/206269a0 5836317

[43] Kawamura, T., Liu, D., Towle, J., Kageyama, R., Tsukahara, N., Wakabayashi, T. et al. (2003) Anti-angiogenesis effects of borrelidin are mediated through distinct pathways: threonyl-tRNA synthetase and caspases are independently involved in suppression of proliferation and induction of apoptosis in endothelial cells. J. Antibiot. 56, 709–715 10.7164/antibiotics.56.709 14563161

[44] Wilkinson, B., Gregory, M.A., Moss, S.J., Carletti, I., Sheridan, R.M., Kaja, A. et al. (2006) Separation of anti-angiogenic and cytotoxic activities of borrelidin by modification at the C17 side chain. Bioorg. Med. Chem. Lett. 16, 5814–5817 10.1016/j.bmcl.2006.08.073 16962775

[45] Mirando, A.C., Fang, P., Williams, T.F., Baldor, L.C., Howe, A.K., Ebert, A.M. et al. (2015) Aminoacyl-tRNA synthetase dependent angiogenesis revealed by a bioengineered macrolide inhibitor. Sci. Rep. 5, 13160 10.1038/srep13160 26271225 PMC4536658

[46] Park, S.G., Ewalt, K.L. and Kim, S. (2005) Functional expansion of aminoacyl-tRNA synthetases and their interacting factors: new perspectives on housekeepers. Trends Biochem. Sci. 30, 569–574 10.1016/j.tibs.2005.08.004 16125937

[47] Williams, T.F., Mirando, A.C., Wilkinson, B., Francklyn, C.S. and Lounsbury, K.M. (2013) Secreted threonyl-tRNA synthetase stimulates endothelial cell migration and angiogenesis. Sci. Rep. 3, 1317 10.1038/srep01317 23425968 PMC3578223

[48] Cao, Z., Wang, H., Mao, X. and Luo, L. (2016) Noncanonical function of threonyl-tRNA synthetase regulates vascular development in zebrafish. Biochem. Biophys. Res. Commun. 473, 67–72 10.1016/j.bbrc.2016.03.051 26993167

[49] Son, K., You, J.S., Yoon, M.S., Dai, C., Kim, J.H., Khanna, N. et al. (2019) Nontranslational function of leucyl-tRNA synthetase regulates myogenic differentiation and skeletal muscle regeneration. J. Clin. Invest. 129, 2088–2093 10.1172/JCI122560 30985292 PMC6486340

[50] Dai, C., Reyes-Ordonez, A., You, J.-S. and Chen, J. (2021) A non-translational role of threonyl-tRNA synthetase in regulating JNK signaling during myogenic differentiation. FASEB J. 35, e21948 10.1096/fj.202101094R 34569098 PMC10226677

[51] Andreucci, J., Grant, D., Cox, D., Tomc, L., Prywes, R., Goldhamer, D. et al. (2002) Composition and function of AP-1 transcription complexes during muscle cell differentiation. J. Biol. Chem. 277, 16426–16432 10.1074/jbc.M110891200 11877423

[52] Lessard, S.J., MacDonald, T.L., Pathak, P., Han, M.S., Coffey, V.G., Edge, J. et al. (2018) JNK regulates muscle remodeling via myostatin/SMAD inhibition. Nat. Commun. 9, 3030–3044 10.1038/s41467-018-05439-3 30072727 PMC6072737

[53] Lo, W.S., Gardiner, E., Xu, Z., Lau, C.F., Wang, F., Zhou, J.J. et al. (2014) Human tRNA synthetase catalytic nulls with diverse functions. Science 345, 328–332 10.1126/science.1252943 25035493 PMC4188629

[54] Lee, E.-Y., Kim, S. and Kim, M.H. (2018) Aminoacyl-tRNA synthetases, therapeutic targets for infectious diseases. Biochem. Pharmacol. 154, 424–434 10.1016/j.bcp.2018.06.009 29890143 PMC7092877

[55] Nie, A., Sun, B., Fu, Z. and Yu, D. (2019) Roles of aminoacyl-tRNA synthetases in immune regulation and immune diseases. Cell Death Dis. 10, 901 10.1038/s41419-019-2145-5 31780718 PMC6883034

[56] Jung, H.-J., Park, S.-H., Cho, K.-M., Jung, K.I., Cho, D. and Kim, T.S. (2020) Threonyl-tRNA synthetase promotes T helper type 1 cell responses by inducing dendritic cell maturation and IL-12 production via an NF-κB pathway. Front. Immunol. 11, 571959 10.3389/fimmu.2020.571959 33178197 PMC7592646

[57] Galindo-Feria, A.S., Notarnicola, A., Lundberg, I.E. and Horuluoglu, B. (2022) Aminoacyl-tRNA synthetases: on anti-synthetase syndrome and beyond. Front. Immunol. 13, 866087 10.3389/fimmu.2022.866087 35634293 PMC9136399

[58] Kim, S.-M., Park, S., Hwang, S.-H., Lee, E.-Y., Kim, J.-H., Lee, G.S. et al. (2023) Secreted *Akkermansia muciniphila* threonyl-tRNA synthetase functions to monitor and modulate immune homeostasis. Cell Host Microbe 31, 1021–1037.e10 10.1016/j.chom.2023.05.007 37269833

[59] Liu, G.Y. and Sabatini, D.M. (2020) mTOR at the nexus of nutrition, growth, ageing and disease. Nat. Rev. Mol. Cell Biol. 21, 183–203 10.1038/s41580-019-0199-y 31937935 PMC7102936

[60] Saxton, R.A. and Sabatini, D.M. (2017) mTOR signaling in growth, metabolism, and disease. Cell 168, 960–976 10.1016/j.cell.2017.02.004 28283069 PMC5394987

[61] Zoncu, R., Efeyan, A. and Sabatini, D.M. (2011) mTOR: from growth signal integration to cancer, diabetes and ageing. Nat. Rev. Mol. Cell Biol. 12, 21–35 10.1038/nrm3025 21157483 PMC3390257

[62] Bonfils, G., Jaquenoud, M., Bontron, S., Ostrowicz, C., Ungermann, C. and De Virgilio, C. (2012) Leucyl-tRNA synthetase controls TORC1 via the EGO complex. Mol. Cell 46, 105 10.1016/j.molcel.2012.02.009 22424774

[63] Han, J.M., Jeong, S.J., Park, M.C., Kim, G., Kwon, N.H., Kim, H.K. et al. (2012) Leucyl-tRNA synthetase is an intracellular leucine sensor for the mTORC1-signaling pathway. Cell 149, 410–424 10.1016/j.cell.2012.02.044 22424946

[64] Wolfson, R.L., Chantranupong, L., Saxton, R.A., Shen, K., Scaria, S.M., Cantor, J.R. et al. (2016) Sestrin2 is a leucine sensor for the mTORC1 pathway. Science 351, 43–48 10.1126/science.aab2674 26449471 PMC4698017

[65] Jewell, J.L., Kim, Y.C., Russell, R.C., Yu, F.X., Park, H.W., Plouffe, S.W. et al. (2015) Metabolism. differential regulation of mTORC1 by leucine and glutamine. Science 347, 194–198 10.1126/science.1259472 25567907 PMC4384888

[66] Wang, S., Tsun, Z.-Y., Wolfson, R.L., Shen, K., Wyant, G.A., Plovanich, M.E. et al. (2015) Lysosomal amino acid transporter SLC38A9 signals arginine sufficiency to mTORC1. Science 347, 188–194 10.1126/science.1257132 25567906 PMC4295826

[67] Chantranupong, L., Scaria, S.M., Saxton, R.A., Gygi, M.P., Shen, K., Wyant, G.A. et al. (2016) The CASTOR proteins are arginine sensors for the mTORC1 pathway. Cell 165, 153–164 10.1016/j.cell.2016.02.035 26972053 PMC4808398

[68] Gu, X., Orozco, J.M., Saxton, R.A., Condon, K.J., Liu, G.Y., Krawczyk, P.A. et al. (2017) SAMTOR is an S-adenosylmethionine sensor for the mTORC1 pathway. Science 358, 813–818 10.1126/science.aao3265 29123071 PMC5747364

[69] Jung, J.W., Macalino, S.J.Y., Cui, M., Kim, J.E., Kim, H.J., Song, D.G. et al. (2019) Transmembrane 4 L six family member 5 senses arginine for mTORC1 signaling. Cell Metab. 29, 1306–1319.e7 10.1016/j.cmet.2019.03.005 30956113

[70] Sancak, Y., Peterson, T.R., Shaul, Y.D., Lindquist, R.A., Thoreen, C.C., Bar-Peled, L. et al. (2008) The Rag GTPases bind raptor and mediate amino acid signaling to mTORC1. Science 320, 1496–1501 10.1126/science.1157535 18497260 PMC2475333

[71] Kim, S.-H., Choi, J.-H., Wang, P., Go, C.D., Hesketh, G.G., Gingras, A.-C. et al. (2021) Mitochondrial threonyl-tRNA synthetase TARS2 is required for threonine-sensitive mTORC1 activation. Mol. Cell 81, 398–407.e4 10.1016/j.molcel.2020.11.036 33340489

[72] Kim, S., You, S. and Hwang, D. (2011) Aminoacyl-tRNA synthetases and tumorigenesis: more than housekeeping. Nat. Rev. Cancer 11, 708–718 10.1038/nrc3124 21941282

[73] Wang, J., Vallee, I., Dutta, A., Wang, Y., Mo, Z., Liu, Z. et al. (2020) Multi-omics database analysis of aminoacyl-tRNA synthetases in cancer. Genes (Basel) 11, 1384 10.3390/genes11111384 33266490 PMC7700366

[74] Sung, Y., Yoon, I., Han, J.M. and Kim, S. (2022) Functional and pathologic association of aminoacyl-tRNA synthetases with cancer. Exp. Mol. Med. 54, 553–566 10.1038/s12276-022-00765-5 35501376 PMC9166799

[75] Jakubaszek, M., Kwiatkowska, B. and Maślińska, M. (2015) Polymyositis and dermatomyositis as a risk of developing cancer. Reumatologia 53, 101–105 10.5114/reum.2015.51510 27407235 PMC4847280

[76] Lánczky, A. and Győrffy, B. (2021) Web-based survival analysis tool tailored for medical research (KMplot): development and implementation. J. Med. Internet Res. 23, e27633 10.2196/27633 34309564 PMC8367126

[77] Jeong, S.J., Kim, J.H., Lim, B.J., Yoon, I., Song, J.A., Moon, H.S. et al. (2018) Inhibition of MUC1 biosynthesis via threonyl-tRNA synthetase suppresses pancreatic cancer cell migration. Exp. Mol. Med. 50, e424 10.1038/emm.2017.231 29328069 PMC5799795

[78] Wellman, T.L., Eckenstein, M., Wong, C., Rincon, M., Ashikaga, T., Mount, S.L. et al. (2014) Threonyl-tRNA synthetase overexpression correlates with angiogenic markers and progression of human ovarian cancer. BMC Cancer 14, 620 10.1186/1471-2407-14-620 25163878 PMC4155084

[79] Tian, H., Yan, H., Zhang, Y., Fu, Q., Li, C., He, J. et al. (2022) Knockdown of mitochondrial threonyl-tRNA synthetase 2 inhibits lung adenocarcinoma cell proliferation and induces apoptosis. Bioengineered 13, 5190–5204 10.1080/21655979.2022.2037368 35184682 PMC8974053

[80] Chen, Y., Ruan, Z.-R., Wang, Y., Huang, Q., Xue, M.-Q., Zhou, X.-L. et al. (2018) A threonyl-tRNA synthetase-like protein has tRNA aminoacylation and editing activities. Nucleic Acids Res. 46, 3643–3656 10.1093/nar/gky211 29579307 PMC5909460

[81] Zeng, Q.Y., Zhang, F., Zhang, J.H., Hei, Z., Li, Z.H., Huang, M.H. et al. (2023) Loss of threonyl-tRNA synthetase-like protein Tarsl2 has little impact on protein synthesis but affects mouse development. J. Biol. Chem. 299, 104704 10.1016/j.jbc.2023.104704 37059185 PMC10200997

[82] Kim, K., Park, S.J., Na, S., Kim, J.S., Choi, H., Kim, Y.K. et al. (2013) Reinvestigation of aminoacyl-tRNA synthetase core complex by affinity purification-mass spectrometry reveals TARSL2 as a potential member of the complex. PLoS One 8, e81734 10.1371/journal.pone.0081734 24312579 PMC3846882

[83] Zhou, X.L., Chen, Y., Zeng, Q.Y., Ruan, Z.R., Fang, P. and Wang, E.D. (2019) Newly acquired N-terminal extension targets threonyl-tRNA synthetase-like protein into the multiple tRNA synthetase complex. Nucleic Acids Res. 47, 8662–8674 10.1093/nar/gkz588 31287872 PMC6794377

[84] Park, S.J., Ahn, H.S., Kim, J.S. and Lee, C. (2015) Evaluation of multi-tRNA synthetase complex by multiple reaction monitoring mass spectrometry coupled with size exclusion chromatography. PLoS One 10, e0142253 10.1371/journal.pone.0142253 26544075 PMC4636271

